# Novel Cesium Resistance Mechanism of Alkaliphilic Bacterium Isolated From Jumping Spider Ground Extract

**DOI:** 10.3389/fmicb.2022.841821

**Published:** 2022-03-08

**Authors:** Takahiro Koretsune, Yoshiki Ishida, Yuri Kaneda, Eri Ishiuchi, Miyu Teshima, Nanami Marubashi, Katsuya Satoh, Masahiro Ito

**Affiliations:** ^1^Graduate School of Life Sciences, Toyo University, Oura-gun, Japan; ^2^Faculty of Life Sciences, Toyo University, Oura-gun, Japan; ^3^Department of Radiation-Applied Biology Research, Takasaki Advanced Radiation Research Institute, Quantum Beam Science Research Directorate, National Institutes for Quantum Science and Technology, Takasaki, Japan; ^4^Bio-Nano Electronics Research Center, Toyo University, Kawagoe, Japan; ^5^Bio-Resilience Research Project (BRRP), Toyo University, Oura-gun, Japan

**Keywords:** cesium-resistant microorganisms, *Microbacterium*, alkaliphilic, mutant, whole-genome sequencing

## Abstract

The radionuclide isotopes (^134^Cs and ^137^Cs) of Cesium (Cs), an alkali metal, are attracting attention as major causes of radioactive contamination. Although Cs^+^ is harmful to the growth of plants and bacteria, alkaliphilic bacterium *Microbacterium* sp. TS-1, isolated from a jumping spider, showed growth even in the presence of 1.2 M CsCl. The maximum concentration of Cs^+^ that microorganisms can withstand has been reported to be 700 mM till date, suggesting that the strain TS-1 is resistant to a high concentration of Cs ions. Multiple reports of cesium ion-resistant bacteria have been reported, but the detailed mechanism has not yet been elucidated. We obtained Cs ion-sensitive mutants and their revertant mutants from strain TS-1 and identified a Cs ion resistance-related gene, *MTS1_00475*, by performing SNP analysis of the whole-genome sequence data. When exposed to more than 200 mM Cs^+^ concentration, the intracellular Cs^+^ concentration was constantly lowered by MTS1_00475, which encodes the novel low-affinity Cs^+^/H^+^ antiporter. This study is the first to clarify the mechanism of cesium resistance in unexplained cesium-resistant microorganisms. By clarifying the new cesium resistance mechanism, it can be expected to be used as a bioremediation tool for treating radioactive Cs^+^ contaminated water.

## Introduction

Cesium (Cs) is an alkali metal, and ^134^Cs and ^137^Cs are radioactive isotopes. Radioactive ^137^Cs is produced from nuclear power generation waste and has attracted considerable attention as a major causative agent of radioactive contamination (Buesseler et al., 2012). The physicochemical properties of Cs^+^ are similar to those of potassium ions (K^+^; Avery, 1995). Studies have reported that Cs^+^ uptake into cells *via* the K^+^ uptake system is localized in the cell membrane of plants and bacteria and high concentrations of Cs^+^ are toxic (Perkins and Gadd, 1995; Hampton et al., 2004), because of decreased intracellular K^+^ concentration due to the influx of Cs^+^ into the cell (Bossemeyer et al., 1989; Jung et al., 2001). Since intracellular K^+^ plays an important role in various physiological functions, such as maintenance of intracellular osmotic pressure (Jung et al., 2001), formation of membrane potential, and regulation of enzyme activity, when Cs^+^ influx and intracellular K^+^ are insufficient (Bossemeyer et al., 1989; Jung et al., 2001). In *Escherichia coli*, as it lacks a Cs^+^ efflux system, Cs^+^ influx into cells occurs through the Kup system, the main K^+^ uptake system (Bossemeyer et al., 1989), resulting in an increased intracellular Cs^+^ concentration. In contrast, K^+^ is extracellularly effluxed by the K^+^ efflux system resulting in only K^+^ excretion from inside the cell and accumulation of Cs^+^ inside the cell. The decrease in intracellular K^+^ concentration inhibited the growth of *E. coli* (Bossemeyer et al., 1989). In addition, the expression of the *kdpFABC* operon, which encodes a high-affinity K^+^ uptake system, is induced to compensate for the decrease in intracellular K^+^ concentration (Jung et al., 2001).

Several Cs^+^-resistant microorganisms have been reported (Bossemeyer et al., 1989; Buesseler et al., 2012; Kato et al., 2016; Swer et al., 2016). *Rhodococcus qingshengii* CS98 and *Arthrobacter* sp. KMSZP6, a cesium-accumulating bacterium, is used for bioremediation in radioactive Cs^+^-contaminated environments (Takei et al., 2014; Swer et al., 2016). Other reported Cs^+^-resistant bacteria, *Flavobacterium* sp. 200CS-4 (Kato et al., 2016), *Serratia* sp. Cs60-2 (Dekker et al., 2014), *Yersinia* sp. Cs67-2 (Dekker et al., 2014), and *Bacillus* sp. C-700 (Zhang et al., 2021) showed CsCl resistance at 200, 300, 500, and 700 mM, respectively. *Flavobacterium* sp. 200CS-4 was isolated from forest soil in Hokkaido, and the intracellular Cs^+^ concentration was lower than that of the outer environment (Kato et al., 2016). However, the underlying mechanism has not yet been clarified. *Serratia* sp. Cs60-2 and *Yersinia* sp. Cs67-2 have been isolated from the nuclear fuel reservoir in Cambria, United States, and both have Cs^+^ resistance not found in related species (Dekker et al., 2014). Therefore, these bacteria possess Cs^+^ resistance properties; however, the mechanism has not been clarified. *Arthrobacter* sp. KMSZP6 was isolated from an untouched uranium deposit in India, and when exposed to a Cs^+^-containing solution, it accumulated Cs^+^ in the cells, and its dry weight became approximately three times that of the bacterium before Cs^+^ treatment (Swer et al., 2016). Therefore, *Arthrobacter* sp. KMSZP6 can be used for bioremediation, such as decontaminating Cs^+^-contaminated water; however, Cs^+^ accumulation and the Cs^+^ resistance mechanisms in cells have not been clarified. *Bacillus* sp. Cs-700 was isolated from sediments in the South China Sea, and related species were identified by 16S rRNA analysis and whole-genome analysis, but the Cs^+^ resistance mechanism is unknown (Zhang et al., 2021). As described above, although multiple Cs^+^-resistant bacteria have been reported, the Cs^+^ resistance mechanism and proteins involved have not been clarified and identified, respectively.

In 2012, we isolated the *Microbacterium* sp. TS-1 (referred to as TS-1) from jumping spiders (Fujinami et al., 2013). Strain TS-1 is a facultative alkaliphilic bacterium with a growth pH range of 6.0–10.0 and an optimum growth pH range of 8.0–9.0. Strain TS-1 was closely related to *Microbacterium arborescens* based on 16S ribosomal DNA phylogenetic analysis. The whole-genome sequence of strain TS-1 was completed in 2013 and registered in the DNA Data Bank of Japan (DDBJ) (accession number for the genomic information of the TS-1 strain is BASQ01000000). In this study, strain TS-1 was a high-concentration Cs^+^-resistant bacterium that could withstand up to 1.2 M CsCl. Therefore, strain TS-1 is expected to exhibit a unique Cs^+^ resistance mechanism. Two major hypotheses regarding the Cs^+^ resistance mechanism of strain TS-1 have been developed. The first is the mechanism that keeps the intracellular Cs^+^ concentration low by effluxing the intracellular Cs^+^ extracellularly by membrane transporters. The second mechanism is that the protein that adsorbs Cs^+^ influx into the cell exists and reduces the free Cs^+^.

In this study, to verify our hypothesis of the Cs^+^ resistance mechanism of strain TS-1, we first obtained Cs^+^-sensitive mutants by chemical treatment with ethyl methanesulfonate (EMS). Several Cs^+^-resistant revertant strains were isolated from each Cs^+^-sensitive mutant by spontaneous mutagenesis. Subsequently, whole-genome sequence analysis of the mutants was performed using a next-generation sequencer. Finally, the mutation sites of the Cs^+^-sensitive mutants and their revertant mutants were comparatively analyzed to identify the Cs^+^ resistance-related gene candidates. We believe that our findings will significantly improve the understanding of the mechanisms of bacterial adaptation to cesium ions.

## Materials and Methods

### Bacterial Strains and Plasmids

The bacterial strains and plasmids used in the present study are listed in Table 1, and the primers used in our investigation are available upon request. The wild-type strain was alkaliphilic *Microbacterium* sp. TS-1, with its whole-genome previously sequenced (Fujinami et al., 2013).

**Table 1:** Bacterial strains and plasmids used in this study.

Strains	Genotype	References
*Microbacterium* sp. TS-1	Wild type	Fujinami et al., 2013
Mut3	Cs^+^-sensitive mutant from TS-1	This study
Mut3R	Cs^+^-resistant revertant from Mut3	This study
Mut4	Cs^+^-sensitive mutant from TS-1	This study
Mut4R	Cs^+^-resistant revertant from Mut4	This study
Mut6	Cs^+^-sensitive mutant from TS-1	This study
Mut6R	Cs^+^-resistant revertant from Mut6	This study
Mut8	Cs^+^-sensitive mutant from TS-1	This study
Mut8R	Cs^+^-resistant revertant from Mut8	This study
Mut9	Cs^+^-sensitive mutant from TS-1	This study
Mut9R	Cs^+^-resistant revertant from Mut9	This study
*Escherichia coli*		
W3110	F^−^ *IN(rrnD-rrnE1) rph-1*	*E. coli* Genetic Stock Center
KNabc	*ΔnhaA*, *ΔnhaB*, *ΔchaA*, Kan^+^, Ery^+^, Cam^+^, *supE*, *hsd*, *Δ*5*thi*, *Δ*(*lac-proAB*)/F′, [*traD36*, *proAB*^+^, *lacLq*, *lacZ*, ΔM15]	Nozaki et al., 1998
Mach1™	F^−^, [φ80*lac*ZΔM15], Δ*lac*X74, *hsd*R,(r_K_−, m_K_^+^), Δ*rec*A1398, *end*A1, *ton*A	Thermo Fisher
*Bacillus subtilis*		
BR151MA	*lys3 tripC2* (wild type)	Grundy et al., 1994
*Alkalihalobacillus pseudofirmus* OF4	Wild type	Guffanti et al., 1986
Plasmid		
pBAD24	Cloning expression vector, P_BAD_ promoter, Ap^R^	Guzman et al., 1995
pBAD-00475	pBAD24 + MST1-00475 (*E. coli* codon-optimized sequence)	This study

### Growth Media and Conditions

*Escherichia coli* and *Bacillus subtilis* strains were grown at 37°C in Luria-Bertani (LB) medium (BD Difco™, New Jersey, United States) and 30 mM Tris medium (Imazawa et al., 2016). *Escherichia coli* strains, Mach1™ (Thermo Fisher) and KNabc (three major Na^+^/H^+^ antiporters-deficient; Nozaki et al., 1998) strains were used for routine genetic manipulations and antiport assay, respectively. The *E. coli* KNabc transformants were grown in LBK medium (10 g tryptone; 5 g yeast extract; 6 g KCl, pH 7.5). *Microbacterium* sp. TS-1 (alkaliphilic in nature) and *Alkalihalobacillus pseudofirmus* OF4 (formerly, *Bacillus pseudofirmus* OF4; Patel and Gupta, 2020) were grown at 37°C in neutral complex medium (NC medium) and 30 mM Tris medium (Tris medium). The Tris medium contained 3.63 g Tris base, 1.47 g citric acid monohydrate, 0.5 g yeast extract, 9 g glucose, 1% (v/v) trace elements (Cohen-Bazire et al., 1957) per liter of deionized water (Imazawa et al., 2016). The pH was adjusted to 8 and 9 using 1 M N-methyl-D-glucamine. The pH was adjusted to 7 using 5 N H_2_SO_4_. Tris medium was used for the resistance test evaluation of monovalent cations because the carry-in of cations can be underestimated. The NC medium contained 15.5 g K_2_HPO_4_, 4.5 g KH_2_PO_4_, 0.05 g MgSO_4_·7H_2_O, 0.34 g citric acid, 5 g peptone, 2 g yeast extract, 5 g glucose, and 11.7 g NaCl per liter of deionized water (Aono et al., 1999). The final pH was adjusted to the desired value by adding KOH or H_2_SO_4_ as needed (Fujinami et al., 2011). These media were solidified by adding 1.5% (w/v) agar when necessary. The medium was supplemented with kanamycin (25 μg/ml) or ampicillin (100 μg/ml) when antibiotics were required for growth selection. Cells were grown at 37°C with shaking and monitored by measuring the optical density at 600 nm (OD_600_) using a spectrophotometer.

### Growth Analysis of Cs-Resistant TS-1 Strain

Strain TS-1 was grown in Tris medium (pH 8) containing various CsCl concentrations. *Escherichia coli* W3110 and *B. subtilis* BR151MA were used as control strains. A similar experiment was performed using NC medium (pH 8). *Alkalihalobacillus pseudofirmus* OF4 (OF4) was used as a control for the alkaliphilic bacterium. Single colonies of strains TS-1, *E. coli*, *B. subtilis*, and OF4 were inoculated into CsCl-free precultures and reciprocally shake-cultured at 200 rpm for 16 h at 30°C. Test medium (2 ml) was placed in each 14 ml culture tube, and the preculture was inoculated to an OD_600_ of 0.01. The culture was reciprocally shaken at 200 rpm for 24 h at 30°C. The OD_600_ of each culture was measured, and the Cs^+^ resistance of each strain was evaluated. Three independent experiments were performed.

### Intracellular Cs^+^ Concentration of *Escherichia coli* and Strain TS-1

The method for preparing the sample was based on the method of Ito et al. (1997). *Escherichia coli* W3110 and strain TS-1 in the late log phase of growth in LB medium at 30°C were harvested by centrifugation (3,000 × *g*, 4°C, 10 min), resuspended in LB medium containing several concentrations of CsCl, then cultured at 30°C at 200 rpm for 1 h. Cells were harvested by centrifugation (3,000 *g*, 4°C, 10 min), washed in 300 mM sucrose twice, and then resuspended in 2 ml 5% TCA (w/v) solution, treated at 100°C for 10 min, and then centrifuged (3,000 × *g*, 4°C, 10 min). One milliliter of the supernatant was collected and Cs^+^ concentration was measured. An atomic absorption spectroscope iCE3400 (Thermo Fisher Scientific, Massachusetts, United States) was used for the measurement.

### Examination of Optimum Conditions for Chemical Mutation Treatment by EMS of Strain TS-1

A single colony of strain TS-1 grown on a complex agar plate (pH 8.0) was inoculated into 2 ml NC medium (pH 8.0) and reciprocally shaken at 200 rpm for 18 h at 30°C. Then, 50 μl of the culture was inoculated into four 24φ test tubes containing 5 ml NC medium (pH 8.0), and reciprocating shaking culture was performed at 200 rpm until the OD_600_ reached 0.6 at 30°C. Then, the whole culture medium was centrifuged at 9,100 × *g* for 5 min at 4°C. The supernatant was removed, and the cell pellet was resuspended in 5 ml ice-cold 200 mM phosphate buffer (pH 7.0); subsequently, 100 μl, 150 μl, or 200 μl undiluted ethyl methanesulfonate (EMS) (Sigma-Aldrich, United States) was added. No EMS was added to the remaining test tubes. EMS treatment was performed by shaking at 200 rpm for 2 h at 30°C. Sampling was performed in each test tube over time. After the treatment, the whole amount was dispensed into a 15 ml Falcon centrifuge tube and centrifuged at 9,100 × *g* for 5 min at 4°C. After removing the supernatant, the cell pellets were resuspended of in 5 ml ice-cold 200 mM phosphate buffer (pH 7.0) and centrifuged at 9,100 × *g* for 5 min at 4°C. After performing this washing step twice, the supernatant was removed, and the cell pellet was suspended in 5 ml ice-cold 200 mM phosphate buffer (pH 7.0). Each suspension was diluted 10-fold with ice-cold 200 mM phosphate buffer (pH 7.0). This operation was repeated, and the suspension was serially diluted 10^7^ times; each 100 μl dilution sample was plated on NC agar medium (pH 8.0) and incubated at 30°C for 2 days. Two days later, the viable colony count on the plate was calculated to determine the survival rate. The following formula was used to calculate the survival rate:


$$\mathrm{Survival rate}\;\left(\%\right)=\left[\begin{array}{l} \mathrm{Viable cell count}\;\mathrm{at}\;\mathrm{the start of}\;\\ \mathrm{chemical mutagenesis}/\\ \mathrm{Viable cell count}\;2\;h\;\mathrm{after}\;\\ \mathrm{chemical mutagenesis} \end{array}\right]\times 100.$$

### Isolation of Cs^+^-Sensitive Mutants by Chemical Mutation Treatment and Replica Plating Method

The strain TS-1 was cultured by the method described above, the supernatant was removed, the cell pellets were suspended in 5 ml ice-cold 200 mM phosphate buffer (pH 7.0), and 150 μl of the EMS undiluted solution was added. In addition, mutagen treatment was performed by shaking at 200 rpm for 2 h at 30°C. After the treatment, the whole sample was dispensed into a 15 ml Falcon centrifuge tube and centrifuged at 9,100 × *g* for 5 min at 4°C. The supernatant was removed, and the cell pellet was washed thrice with 5 ml ice-cold 200 mM phosphate buffer (pH 7.0). After washing, the supernatant was removed, and the cell pellet was resuspended in 5 ml NC medium; the entire suspension was transferred to a 24φ test tube and reciprocally shaken at 200 rpm for 18 h at 30°C. After culturing, the whole amount was transferred to a 15 ml Falcon centrifuge tube and centrifuged at 9,100 × *g* for 5 min at 4°C. The supernatant was removed, and the cell pellet suspended in 5 ml ice-cold 200 mM phosphate buffer containing 20% glycerol, 100 μl was poured into 1.5 ml tubes, the rest was stored at −80°C. The poured suspension was diluted 10-fold with ice-cold 200 mM phosphate buffer (pH 7.0), which was further diluted 10-fold with 200 mM phosphate buffer (pH 7.0). This operation was repeated, and the suspension was serially diluted 10^7^ times and plated (100 μl each) on 25 NC agar plates and cultured at 30°C for 2 days. Two days later, the colonies were independently transferred from the colony-formed plate onto an NC agar plate containing 100, 200, and 300 mM CsCl by the replica plating method, and incubated at 30°C for 2 days. After culturing, each plate was compared to identify the one in which colony formation was absent with or without CsCl, and four types of composites containing 100 and 200 mM of CsCl in addition to the NC agar medium. Strains exhibiting reproducible Cs^+^ sensitivity were designated candidate Cs^+^-sensitive mutants, and each strain was stored.

### Isolation of Cs^+^-Resistant Revertants From Cs^+^-Sensitive Mutants

Single colony isolation was performed for the isolated Cs^+^-sensitive mutants on NC agar medium (pH 8). The colonies were inoculated into 2 ml NC medium (pH 8.0) and reciprocally shake-cultured at 200 rpm at 30°C for 18 h. The culture (100 μl) was independently plated on NC agar medium (pH 8) containing 200 mM or 400 mM CsCl to obtain spontaneous mutants whose cesium resistance was restored. The same culture diluted 10^7^-fold with 200 mM phosphate buffer (pH 7) was plated onto an NC agar plate (pH 8.0) for measuring the viable cell count of the culture. Each plate was incubated at 30°C for 2 days. The number of colonies formed on the plate was counted, the viable cell count and the reversion mutation rate were calculated, and the Cs^+^-resistant revertants were isolated.

### Cs^+^ Resistance Growth Test of Cs^+^-Sensitive Mutants and Cs^+^-Resistant Revertant Mutants

Each TS-1 mutant was isolated from a single colony growing on an NC agar plate (pH 8.0). Each mutant strain colony was inoculated into a 14 ml culture tube containing 2 ml NC medium (pH 8.0) and reciprocally shake-cultured at 200 rpm at 30°C for 18 h. The culture broth was used as the preculture broth. Two milliliters of test medium were placed in each 14 ml culture tube, and 10 μl of the preculture was inoculated into each tube. The culture was reciprocally shaken at 200 rpm at 30°C for 16 h. The OD_600_ of each culture was measured, and the Cs^+^ resistance of each strain was evaluated. Three independent experiments were performed.

### Preparation of Chromosomal DNA From TS-1 Mutants

Mut3, Mut3R, and Mut4 mutants isolated from single colonies were inoculated into 2 ml NC medium (pH 8.0) and reciprocally shake-cultured at 30°C and 200 rpm for 18 h. Five hundred microliters of preculture was inoculated into 4.5 ml NC medium (pH 8.0) in a 24φ test tube and reciprocally shake-cultured at 30°C and 200 rpm for 4 h. Then, the whole culture medium was centrifuged at 9,100 × *g* for 5 min at 4°C and the supernatant was removed. Chromosomal DNA was prepared according to the operation manual of the DNeasy Blood & Tissue Kit (QIAGEN, Japan).

### Comparative Analysis of Whole-Genome Sequencing Data

Whole-genome sequencing was performed on chromosomal DNA by Eurofins Genomics K.K using the next-generation sequencer HiSeq X 2 × 150 bp (Illumina, United States). The whole-genome sequence of each mutant strain was subjected to SNP analysis, and the mutation site was extracted. The variant call was analyzed using the following method: variant call: using samtools (ver. 1.6), bases different from the reference were extracted from the mapping results. Filtering: a variant that uses vcfutils.pl. from bcftools (ver. 1.6) and meets the default settings of the called variants. Genes with different mutation sites were selected as candidate Cs^+^ resistance-related genes. The selected Cs^+^ resistance-related candidate gene was identified from the annotation results of the TS-1 strain genome sequence, and it was investigated whether the mutation caused the non-synonymous substitution of amino acids. In addition, mutations were found in genes that overlapped between Cs^+^-sensitive mutant strains, and those in which reverse mutations were found in both revertant mutant strains were selected as promising candidates for Cs^+^ resistance-related genes. Nucleotide sequence data reported are available in the DDBJ Sequenced Read Archive under the accession numbers DRR328004-DRR32806.

### Artificial Synthetic Gene

To express MTS1_00475 in *E. coli*, the *MTS1_00475* gene was optimized for *E. coli* codons using GENEius.^1^ Eurofins Genomics (Tokyo, Japan) artificially synthesized each identified gene. The gene sequence was registered in the DNA Data Bank of Japan (DDBJ). The accession number for the *MTS1_00475* gene is LC654691.

### Alignment of the Cesium Resistance-Related Genes With Homologous Proteins of Several Bacterial Species

The amino acid sequences of the cesium resistance-related candidate genes and several homologs were obtained using the BLASTp algorithm at NCBI.^2^ The selected amino acid residues in the alignment were analyzed using MAFFT ver. 7^3^ (Katoh et al., 2002, 2019). Tree View^4^ was used to create a molecular phylogenetic tree of cesium resistance-related genes and their homologs using the neighbor-joining method (NJ method). From the amino acid sequence of the cesium resistance-related genes, each protein structure was inferred using TMHMM 2.0.^5^

### Construction of Plasmids for Expression of Optimized *Escherichia coli* Codons for *MTS1_00475*

The artificially synthesized DNA fragment of the *MTS1_00475* gene optimized for *E. coli* codons was double digested with restriction enzymes *Bsp*HI and *Xba*I, and pBAD24 double digested with *Nco*I and *Xba*I was ligated with T4 DNA ligase. pBAD24 is an arabinose-inducible expression vector (Guzman et al., 1994). The reaction solution was transformed into *E. coli* Mach1 competent cells. The appropriate amount was spread onto LB agar medium containing 100 μg/ml ampicillin and statically cultured overnight at 37°C. The plasmid was obtained and designated as pBAD-00475.

### Cs^+^ Growth Test of *Escherichia coli* KNabc Transformants

Single colonies of *E. coli* strains KNabc/pBAD24 and KNabc/pBAD-00475 were inoculated into 2 ml LBK medium (pH 7.5) at a final concentration of 100 μg/ml ampicillin and 25 μg/ml kanamycin and reciprocally shake-cultured at 37°C and 200 rpm. The culture broth was used as the preculture broth and 2 ml LBK medium (+0.1% arabinose), and various concentrations (50 mM, 100 mM, 150 mM) of CsCl with a final concentration of 100 μg/ml ampicillin and 25 μg/ml kanamycin were inoculated with 10 μl of the preculture. The culture was reciprocally shaken at 37°C and 200 rpm for 16 h. The OD_600_ of each culture was measured, and the Cs^+^ resistance of each strain was evaluated. Three independent experiments were performed.

### Membrane Preparations From *Escherichia coli* Transformants

Everted membrane vesicles from *E. coli* KNabc transformants were prepared using a standard procedure (Morino et al., 2008). Briefly, 400 ml LBK medium (+0.1% arabinose) was inoculated with a final concentration of 100 μg/ml ampicillin and 25 μg/ml kanamycin, and 1 ml preculture was grown at 37°C and 200 rpm for 16 h. Cells were harvested and then washed with TCDG buffer (10 mM Tris–HCl, pH 8.0; 5 mM MgCl_2_; 10% glycerol; 140 mM choline chloride; 1 mM D-dithiothreitol). Subsequently, the cell suspension was passed through a French press (10,000 psi). After centrifugation at 4°C and 9,100 × *g* for 10 min, the membrane fraction was collected by ultracentrifugation at 14°C and 49,000 × *g* for 60 min and then suspended in fresh TCDG buffer. The membrane fraction was stored at −80°C. Protein content was determined by the Lowry method using lysozyme as a standard (Lowry et al., 1951).

### Cation/H^+^ Antiport Activity in the Everted Membrane Vesicles

*Escherichia coli* KNabc membrane vesicles (66 μg protein) were diluted in 2 ml assay buffer [10 mM bis-tris phosphonate (BTP)-Cl, 5 mM MgCl_2_, 100 mM choline Cl, pH 7.0, 7.5, 8.0, and 8.5] supplemented with 1 μM acridine orange. The assay was initiated by adding succinate to a final concentration of 2.5 mM. After steady-state fluorescence quenching was reached, the cation was added. Finally, 1 mM NH_4_Cl was added to the assay buffer to abolish ΔpH and establish a baseline. Using the baseline, % dequenching, a decrease in the succinate-dependent ΔpH due to cation addition was calculated by tracing the fluorescent changes. The % dequenching from empty vector control vesicles was subtracted from every value from *E. coli* KNabc/pBAD-00475 vesicles. The concentration of cations yielding half-maximal dequenching has been validated as a good estimate of the apparent *K*_m_ of cation/H^+^ antiporters (Swartz et al., 2007). Measurements were conducted using a Hitachi High-Technologies model F-4500 fluorescence spectrophotometer (excitation wavelength: 420 nm, fluorescence wavelength: 500 nm, slit width: 10 nm).

## Results

### Cs^+^ Resistance Growth Test of Strain TS-1

**Figure 1:**
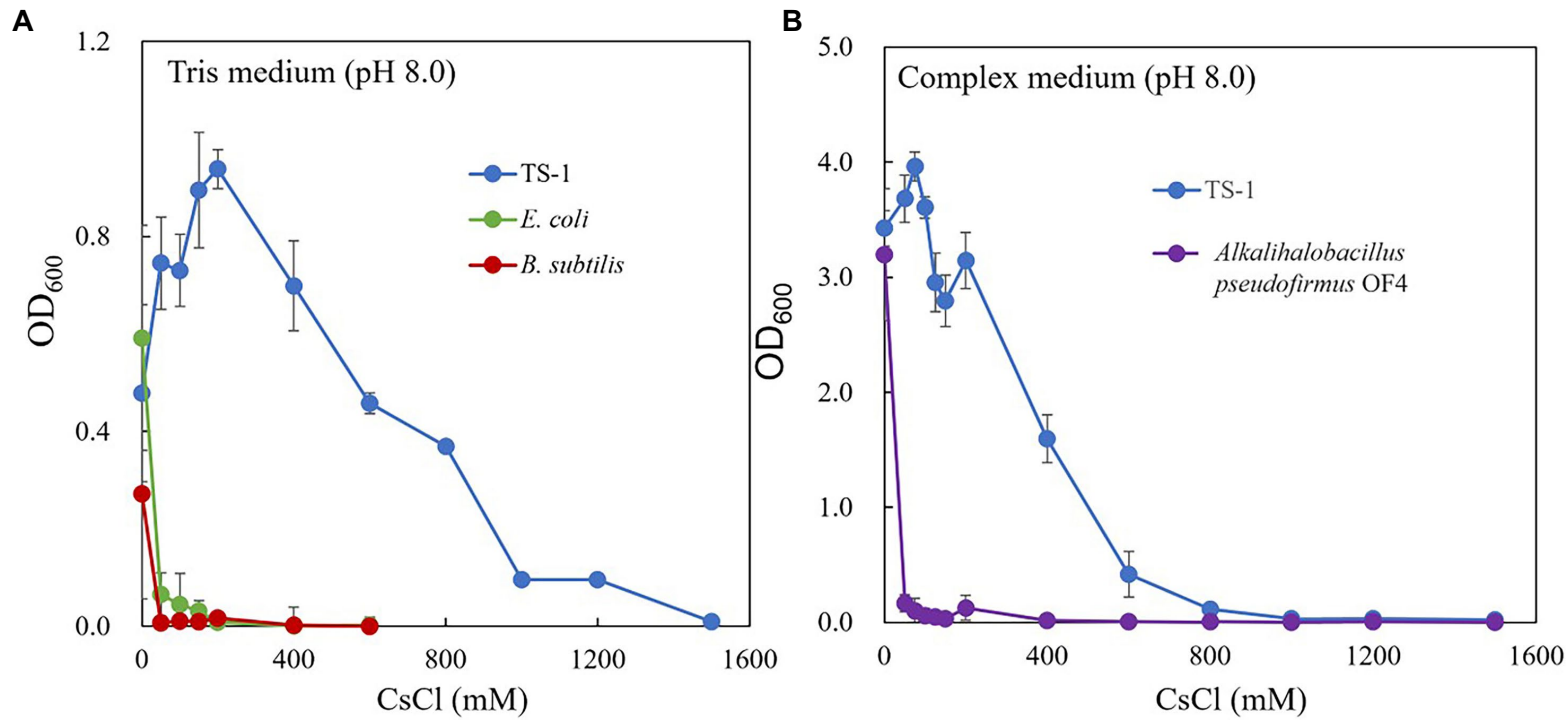
Cs^+^ resistance growth test of strain TS-1. The growth of strain TS-1 on Tris medium (pH 8) was compared to that of *Escherichia coli* and *Bacillus subtilis* at several CsCl concentrations **(A)**. The growth of strain TS-1 on a NC medium (pH 8.0) was compared to that of strain OF4 at several CsCl concentrations **(B)**. Reciprocally shaking culture was performed at 30°C at 200 rpm for 24 h, and the OD_600_ was measured. Three independent experiments were performed. Error bars indicate SD.

The growth of strain TS-1 on Tris medium (pH 8) was compared to that of *E. coli* and *B. subtilis* at several CsCl concentrations (Figure 1A). *Escherichia coli* and *B. subtilis* grew in a medium containing up to 50 mM CsCl, but the TS-1 strain grew up to1200 mM CsCl. The growth of the strain TS-1 in NC medium (pH 8.0) was compared to that of the strain OF4 at several CsCl concentrations (Figure 1B). Strain OF4 showed growth inhibition in the medium containing 50 mM M CsCl, but strain TS-1 was able to grow in media containing up to 600 mM CsCl.

### Intracellular Cs^+^ Concentration of *Escherichia coli* and Strain TS-1

**Figure 2:**
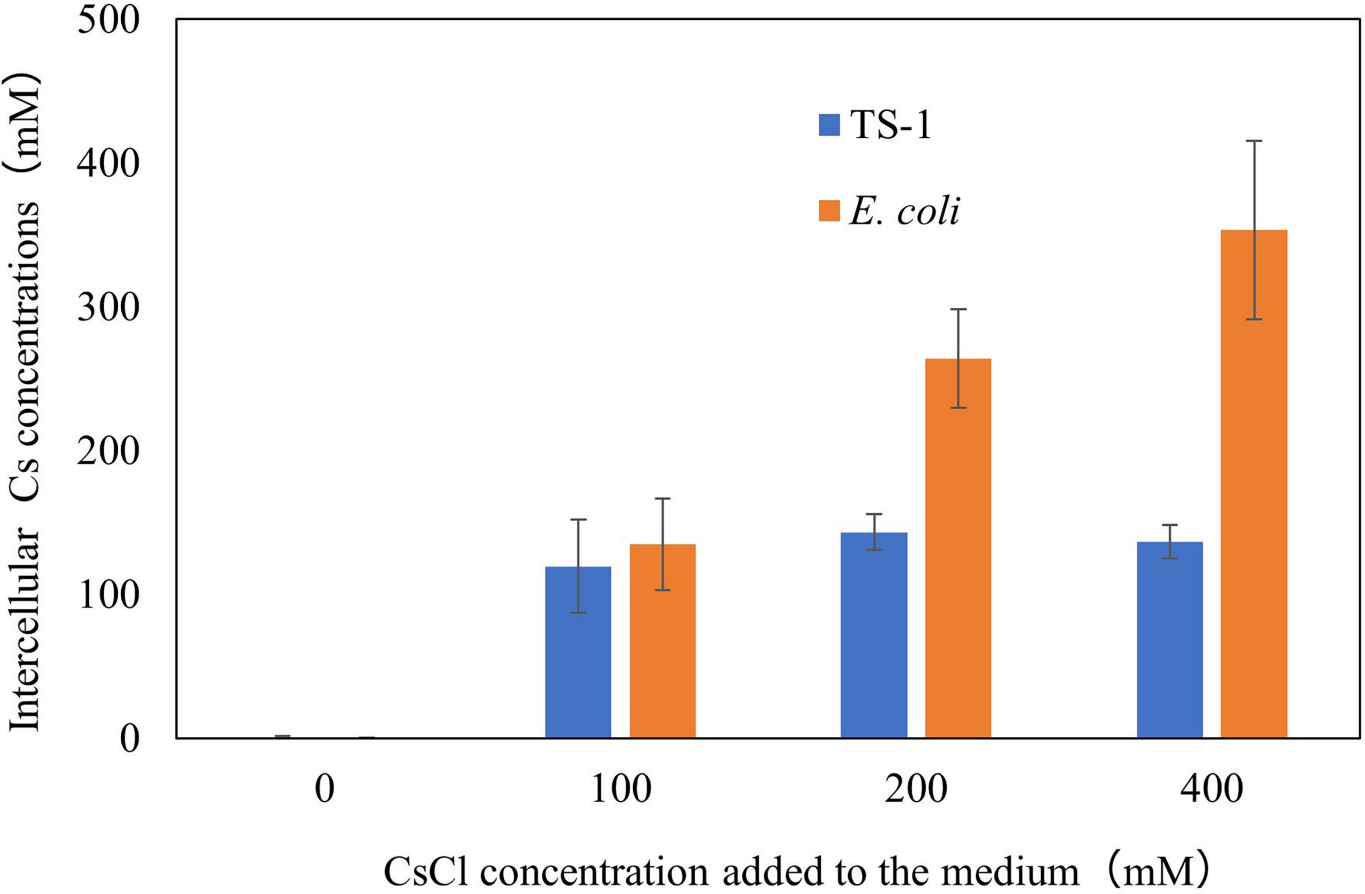
Intracellular Cs^+^ concentration of *E. coli* and strain TS-1 at several CsCl conditions. The details of the experiment are described in the Materials and Methods section. The error bars show the SD for two independent experiments.

The results are shown in Figure 2. *Escherichia coli* increased the intracellular Cs^+^ concentration in correlation with CsCl added to the medium, however, strain TS-1 kept the intracellular Cs^+^ concentration low even when exposed to CsCl of 200 mM or more. Cs^+^-resistant strain *Flavobacterium* sp. 200CS-4 was isolated from forest soil in Hokkaido, and it was clarified by measuring the intracellular Cs^+^ concentration that the intracellular Cs^+^ concentration was kept lower than that in the environment (Kato et al., 2016). However, the mechanism of intracellular Cs^+^ excretion has not been clarified.

### Determination of Chemical Mutagenesis Treatment Conditions by EMS of TS-1 Strain

**Figure 3:**
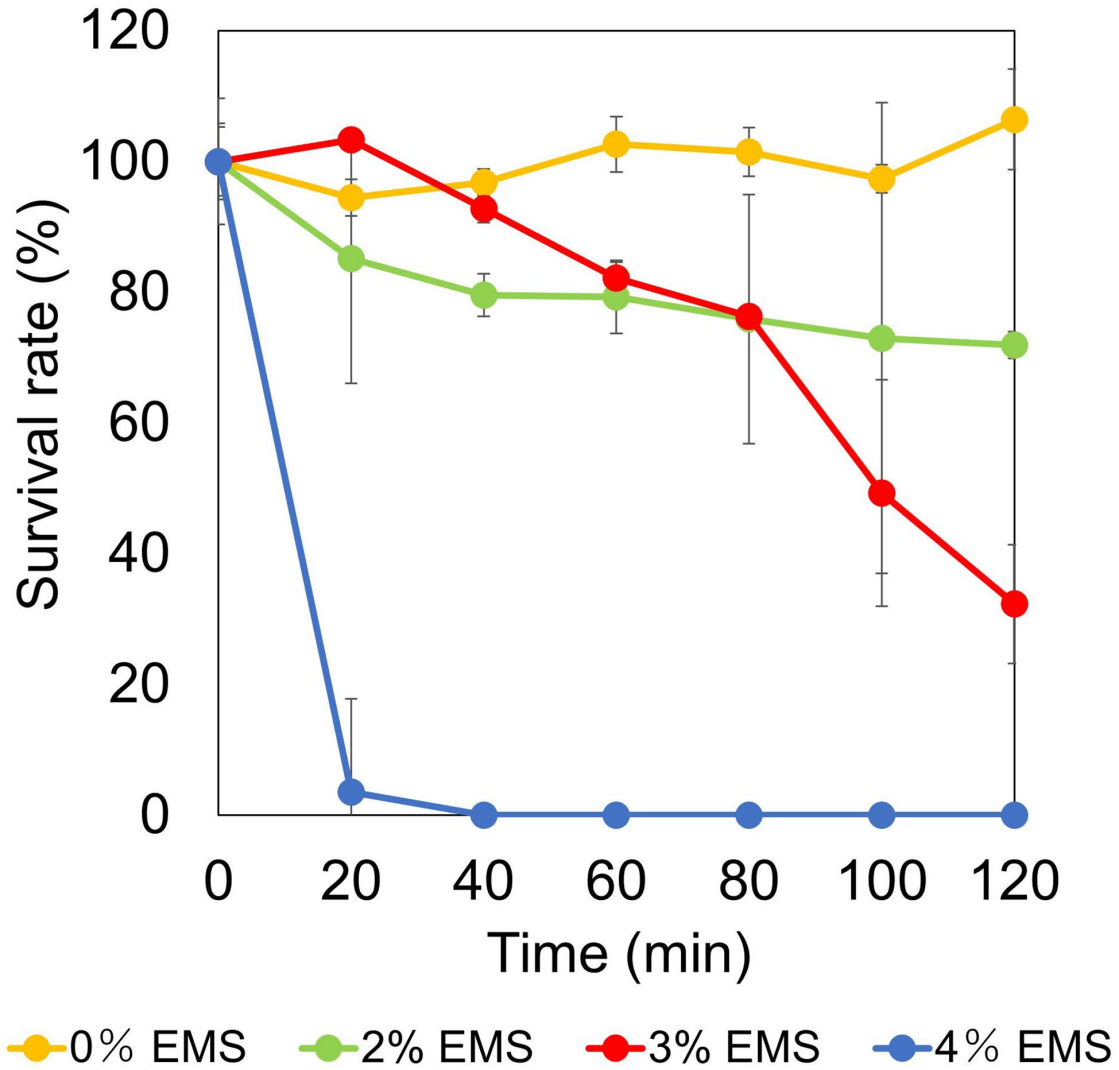
Examination of conditions for chemical mutation treatment by ethyl methanesulfonate (EMS) in strain TS-1. The figure shows the change in survival rate depending on the EMS concentration when the strain TS-1 was subjected to chemical mutation treatment for 120 min. The vertical axis shows the survival rate obtained from the viable cell count, the horizontal axis shows the EMS concentration used for the chemical mutation treatment, and the error bar shows the SD for three independent experiments.

Chemical mutagenesis treatment with different EMS concentrations against strain TS-1 was performed for 120 min to determine the survival rate. The survival rate was 106% when EMS was not administered. In contrast, when EMS was added at a final concentration of 2% (v/v), 3% (v/v), and 4% (v/v), the survival rate changed to 71%, 32%, and 0% (Figure 3). Since the general conditions for chemical mutagenesis treatment were set so that the survival rate was 10%–50%, in the subsequent chemical mutagenesis treatment, experiments were conducted under 3% (v/v) EMS treatment conditions for 2 h.

### Isolation of Cs^+^-Sensitive Mutant Strains by Chemical Mutagenesis Treatment and Replica Plating Method

After performing chemical mutagenesis with 3% (v/v) EMS for 2 h, the cells were cultured until the early stationary phase, spread on plates, and incubated at 30°C for 2 days. Approximately 50,000 colonies were duplicated by the replica plating method, and five candidate mutant strains (Mut3, Mut4, Mut6, Mut8, and Mut9) sensitive to 200 mM Cs^+^ were isolated (Supplementary Figure S1). Strains Mut3, Mut4, and Mut9 did not grow in the medium containing 100 mM CsCl, and Mut6 and Mut8 showed tiny colonies in the medium containing 200 mM CsCl.

### Isolation of Cs^+^ Resistance-Revertant Strains From Each Cs^+^-Sensitive Mutant Strain

The reversion mutation rates of each Cs^+^-sensitive mutant strain were calculated. The reversion mutation rates of strains Mut3, Mut4, Mut6, Mut8, and Mut9 were 5.4 × 10^−10^, 2.0 × 10^−10^, 1.2 × 10^−8^, 2.5 × 10^−8^, and 9.2 × 10^−9^, respectively. In general, the reversion mutation rates are stable at 1.0 × 10^−8^ or less and are unstable at 1.0 × 10^−6^ or more, and all five mutant strains were phenotypically considered stable.

**Figure 4:**
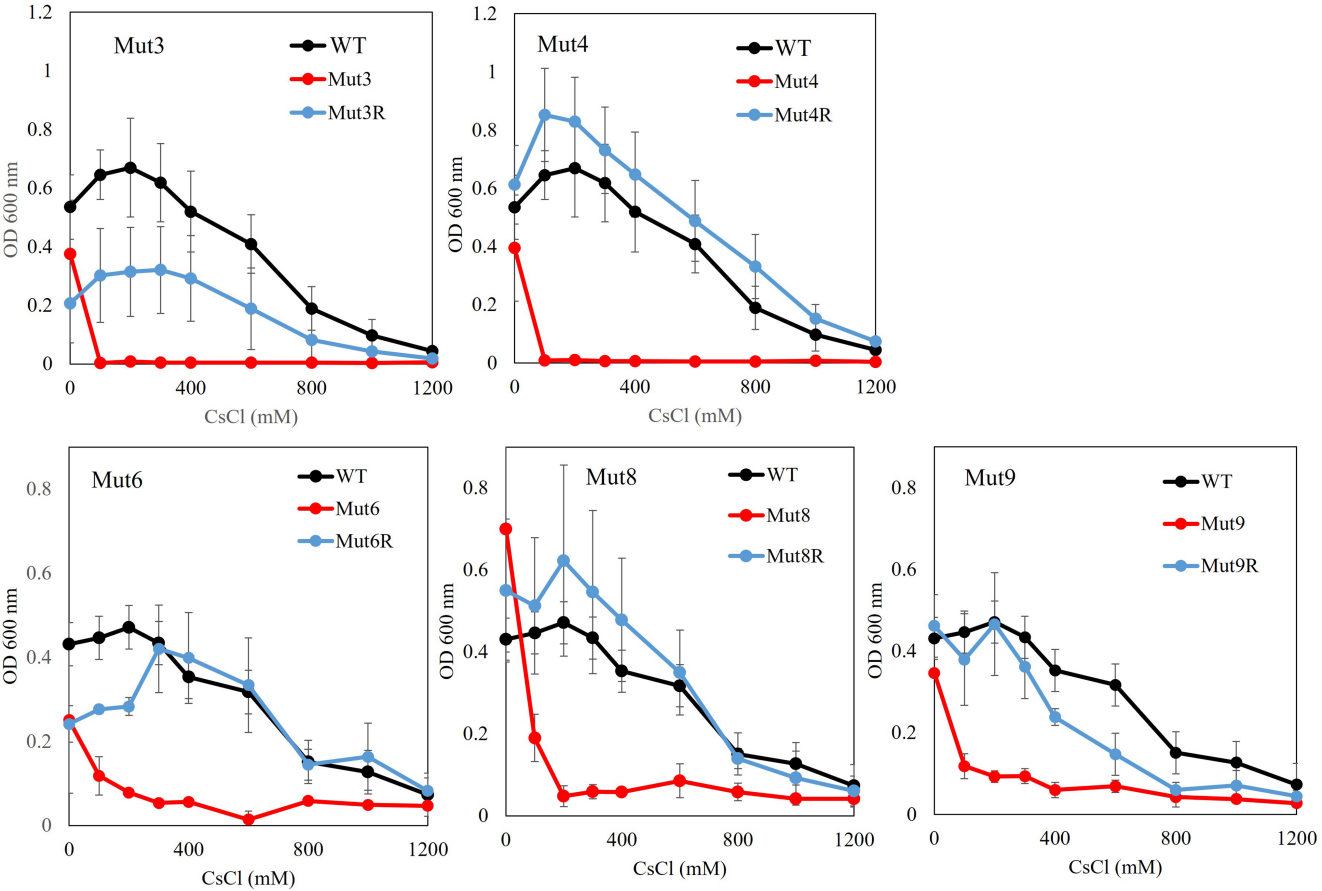
Test to evaluate the Cs^+^ resistance growth of each Cs^+^-sensitive mutant and its revertant mutants. Colonies of each mutant isolated from a single colony were inoculated into 2 ml NC medium (pH 8.0) and reciprocally shake incubated at 200 rpm at 30°C for 18 h. Ten microliters of the preculture were inoculated into 2 ml Tris medium (pH 8.0) with various concentrations of CsCl (100–1,200 mM) and reciprocally shaken at 30°C at 200 rpm for 18 h, and the OD_600_ was measured. Error bars show the SD of three independent experiments.

Growth tests for CsCl were performed for each mutant strain, and the results are shown in Figure 4. All cesium ion-sensitive mutants showed decreased growth compared to the wild type. Each reverted strain regained the same level of cesium resistance as the wild-type strain, except for Mut3R and Mut9R strains, both of which did not recover as much cesium ion resistance as the wild type. However, they recovered resistance to cesium ions compared to the growth of each sensitive mutant.

### Comparative Analysis of Whole-Genome Analysis Results by Next-Generation Sequencing

Whole-genome sequence analysis of strains Mut3 and Mut4, which are Cs^+^-sensitive mutants obtained in the early stage of this project, revealed 145 and 31 mutations, respectively. A common gene encodes a putative permease of the major facilitator superfamily (referred to as MTS1_00475). In addition, a comparison of the genome sequences of strains Mut3 and the revertant mutant Mut3R confirmed that Mut3R had a reversion mutation (Mut3: Q59* → Mut3R: *59Q) on the gene sequence of MTS1_00475. The nucleotide sequence of MTS1_00475 in the revertant was performed. The mutation site (Mut4: W253 Stop codon) in strain Mut4 exhibited intergenic suppression (Stop codon253R) in strain Mut4R. Mutations for strains Mut6, Mut8, and Mut9, isolated after strains Mut3 and Mut4, were also identified in *MTS1_00475* by analyzing the *MTS1_00475* region nucleotide sequences. In addition, the analysis of the nucleotide sequences of the *MTS1_00475* region for strains Mut6R, Mut8R, and Mut9R confirmed that the mutant site had reverted to the wild type and that intergenic suppression had occurred for Mut8R and Mut9R. The details of the mutant strains are summarized in Table 2.

**Table 2:** The reversion mutation rates of each Cs^+^-sensitive mutant strain proteins encoded by genes with revertant mutations in each revertant mutant.

Mutants	The frequency of Cs^+^-resistant revertant strains	Amino acid mutation site (Cs^+^-sensitive mutant → revertant mutant)	Accession number
Mut3R	5.4 × 10^−10^	**MTS1_00475** (Permease of the major facilitator superfamily) (Q59* → *59Q) (true reversion)	BASQ01000001.1 505359–506906 (minus strand)
		MTS1-02327 50S ribosomal protein L16 (T122I → I122T) (true reversion)	BASQ01000001.1 2480985–2481404 (minus strand)
Mut4R	2.0 × 10^−10^	**MTS1_00475** (W253* → *253R) (Intragenic suppression)	BASQ01000001.1 505359–506906 (minus strand)
Mut6R	1.2 × 10^−8^	**MTS1_00475** (G164D → D164G) (true reversion)	BASQ01000001.1 505359–506906 (minus strand)
MutR8R	2.5 × 10^−8^	**MTS1_00475** (W19* → *19Y) (Intragenic suppression)	BASQ01000001.1 505359–506906 (minus strand)
Mut9R	9.2 × 10^−9^	**MTS1_00475** (W177* → *177R) (Intragenic suppression)	BASQ01000001.1 505359–506906 (minus strand)

### Bioinformatics Analysis of MTS1_00475

**Figure 5:**
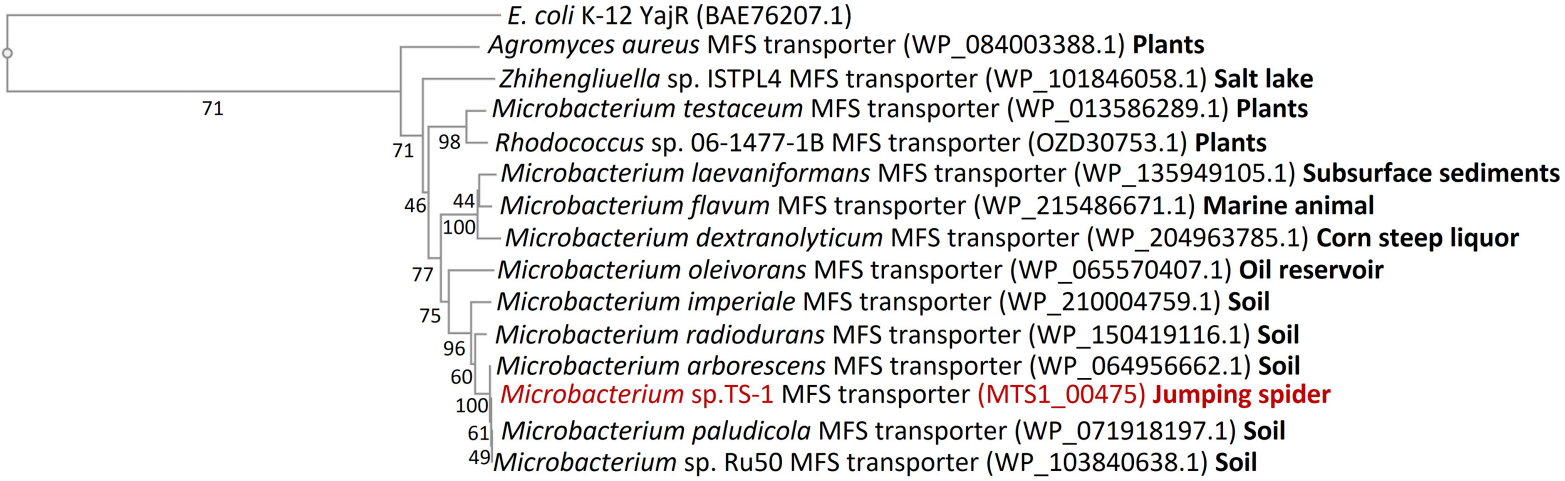
Molecular phylogenetic tree analysis of MTS1_00475. A molecular phylogenetic tree was constructed based on multiple sequence alignment with the homolog of MTS1_00475. The details are described in the Materials and Methods section. MTS1_00475 from *Microbacterium* sp. TS-1 is shown in red. YajR from the major facilitator superfamily (MFS) of transporters of *E. coli* K-12 was used as an outgroup. The number between branches indicates the bootstrap value. The number after the bacterium indicates the GenBank accession number. The locations of isolation of the 13 strains harboring the MTS1_00475 homologs used to create the phylogenetic tree are shown in bold after each accession number.

Multiple sequence alignment of MTS1_00475 and its homologous proteins revealed that MTS1_00475 is widely distributed in the genus *Microbacterium* (Figure 5). In addition, MTS1_00475 showed 99% homology with the major facilitator superfamily (MFS) transporter of *Microbacterium* sp. Ru50 and *Microbacterium paludicola*. Furthermore, homologs were also identified for the MFS transporter of *Rhodococcus* sp. 06-1477-1B, *Zhihengliuella* sp. ISTPL4, and *Agromyces aureus*, and their amino acid identities were 73%, 72%, and 69%, respectively. A molecular phylogenetic tree based on these results is shown in Figure 5. The locations of the homologues of MTS1_00475 of the 13 isolated strains used to create the phylogenetic tree were investigated. The following bacteria and associated locations were identified: three bacteria related to plants (root spheres of plants, potato leaves, and a symptomatic Veronica plant; Morohoshi et al., 2011; Corretto et al., 2016; Savory et al., 2017); two bacteria isolated from the aquatic environment (marine animals like *Dictyopodium moseleyi* and salt lakes; Kageyama et al., 2007; Mishra et al., 2018); five bacteria derived from soil (Collins et al., 1983; Park et al., 2006; Kinegam et al., 2007; Zhang et al., 2010); one bacterium isolated from subsurface sediments (Brown et al., 2012); one bacterium isolated from oil reservoirs (Schippers et al., 2005); one bacterium isolated from Corn steep liquor (Yokota et al., 1993). *Microbacterium* spp. which harbor a protein with high homology to MTS1_00475 were obtained from the soil.

**Figure 6:**
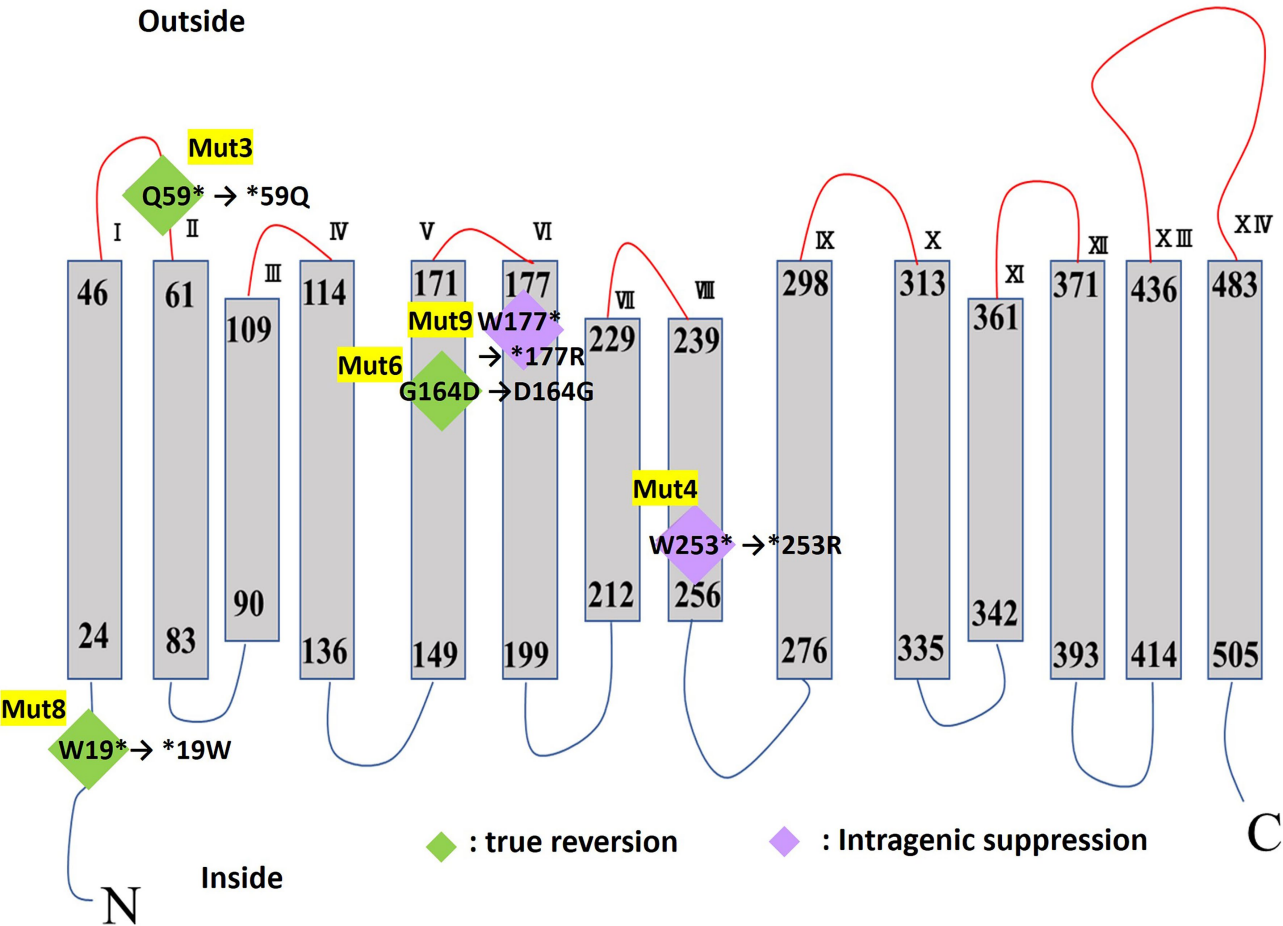
TMHMM transmembrane model of MTS1_00475. The mutation site of each Cs^+^-sensitive mutant strain is shown in diamonds. True reversion is shown in green diamonds, and in purple diamonds, intergenic suppression for mutations in the Cs^+^-resistant revertant mutant strain. The asterisk indicates a stop codon.

MTS1_00475 was predicted to be a 14-transmembrane protein based on protein structure prediction using TMHMM-2.0. A schematic of the predicted secondary structure is shown in Figure 6.

### Measurement of Cs^+^/H^+^ Antiport Activity by Fluorescence Quenching Using Everted Membrane Vesicles

**Figure 7:**
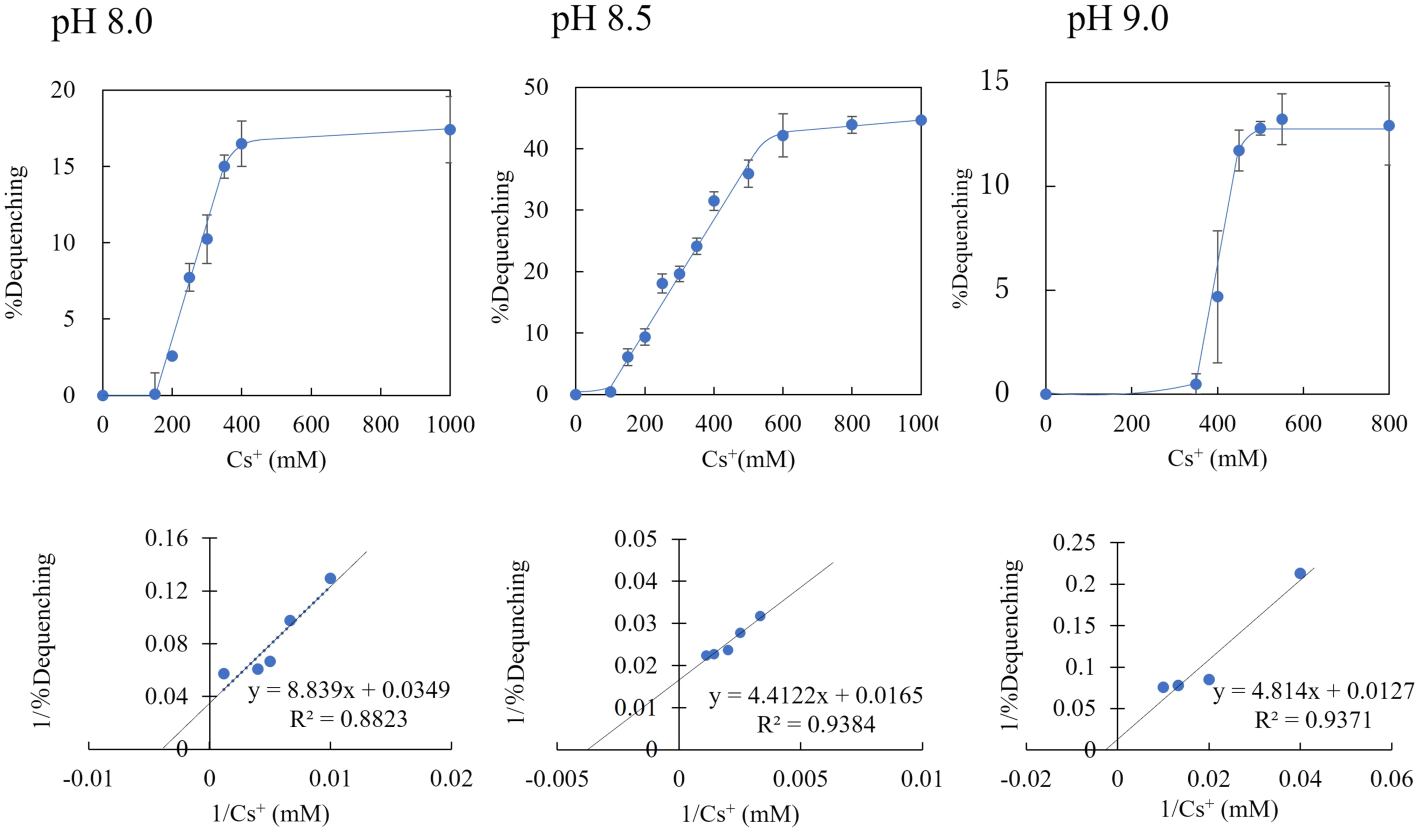
Cs^+^/H^+^ antiport activity of everted membrane vesicle from strain KNabc/pBAD-00475 at pH 8.0, 8.5, and 9.0. The Cs^+^/H^+^ antiport activity of everted membrane vesicles from strain KNabc/pBAD-00475 in each pH buffer was shown when various concentrations of Cs_2_SO_4_ were added. The details are described in the Materials and Methods section. The vertical axis shows antiport activity (% Dequenching), and the horizontal axis shows the Cs^+^ concentration. Error bars show the standard deviation of three independent experiments. In addition, each Lineweaver-Burk plot diagram is shown.

We prepared everted membrane vesicles from KNabc/pBAD-00475 and KNabc/pBAD24 and then measured the Cs^+^/H^+^ antiporter activity (Figure 7). The vesicle sample of KNabc/pBAD-00475 showed no Cs^+^/H^+^ antiport activity in buffers at pH 7.0, 7.5, and Cs^+^/H^+^ antiport activity was measured between pH 8.0 and 9.0. This enzyme was the most active pH 8.5 buffer and showed 44.6% Cs^+^/H^+^ antiporter activity. In addition, the Cs^+^/H^+^ antiport activity was observed when Cs^+^ concentration changed in the buffer solution of pH 8.0–9.0. The apparent *K*_m_ value for Cs^+^ was calculated from the Lineweaver–Burk plot equation and was found to be 250 mM, 370 mM, and 410 mM for pH 8.0, 8.5, and 9.0, respectively (Figure 7), indicating that MTS1_00475 is a low-affinity Cs^+^/H^+^ antiporter. When the antiport activity at pH 8.0 in other monovalent cations was also measured, the apparent *K*_m_ value in Na^+^, K^+^, and Rb^+^ was 1.7, 0.9, and 3.4 mM, respectively (Supplementary Figure S2). These data showed that MTS1_00475 is a membrane protein that transports not only Cs^+^ but also monovalent cations, such as Na^+^, K^+^, and Rb^+^.

### Cs^+^ Resistance Growth Test of *Escherichia coli* KNabc/pBAD-00475

**Figure 8:**
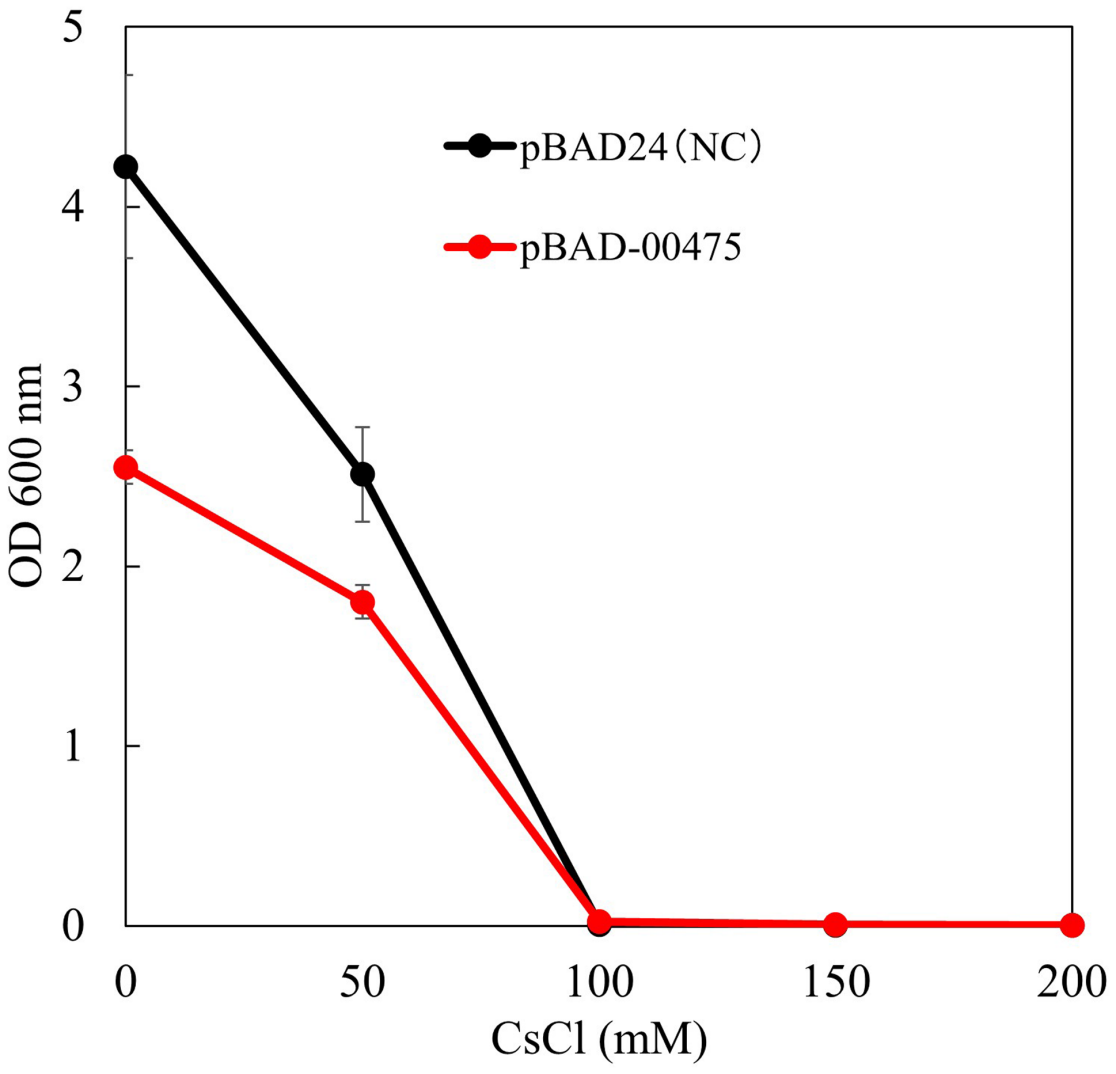
Test to evaluate the Cs^+^ resistance growth of strain KNabc/pBAD-00475. The turbidity of each concentration of CsCl when strain KNabc/pBAD-00475 in LBK medium was cultured for 16 h. Error bars show the standard deviation of three independent experiments. As the negative control, KNabc/pBAD24 was used.

To confirm whether the Cs^+^ resistance of *E. coli* KNabc expressing MST1_00475 was improved, a Cs^+^ resistance growth test was conducted. Figure 8 shows the results of OD_600_ after independently culturing *E. coli* KNabc expressing MST1_00475 in a medium with different CsCl concentrations for 16 h. KNabc/pBAD-00475 and KNabc/pBAD24 (negative control) showed no growth in the presence of 100mM CsCl. The expression of MTS1_00475 confirmed no improvement in Cs^+^ resistance. This result may be related to the low affinity of MTS1_00475 Cs^+^/H^+^ antiport activity.

## Discussion

### High Concentration Cesium Ion Resistance Strain TS-1

In recent years, there have been multiple reports of high-concentration cesium-resistant bacteria. Still, there have been no reports on the mechanism underlying the cesium ion resistance of these bacteria (Bossemeyer et al., 1989; Buesseler et al., 2012; Kato et al., 2016; Swer et al., 2016). *Bacillus* sp. C-700 is most CsCl-resistant bacterium, although strain C-700 did not show growth in a previous study (Zhang et al., 2021). We report that *Microbacterium* sp. strain TS-1, isolated from jumping spider ground extract, can grow in Tris medium (pH 8) containing 1,200 mM CsCl. Under the same growth conditions, *E. coli* and *B. subtilis* showed growth inhibition in the presence of 50 mM CsCl (Figure 1A). Therefore, owing to its resistance to high concentrations of cesium ions, strain TS-1 was expected to harbor unique Cs^+^ resistance mechanisms.

### Isolation of Cesium Ion-Sensitive Strains and Their Revertants, and Identification of Cesium Resistance Genes by Whole-Genome Sequence Analysis

Five Cs^+^-sensitive mutants (Mut3, Mut4, Mut6, Mut8, and Mut9) were isolated through EMS treatment and by using the replica method of strain TS-1 (Supplementary Figure S1). A growth test of the Cs^+^ resistance phenotype of each Cs^+^-sensitive mutant and its revertant strain showed different results (Figure 4). In addition, the reversion mutation rate of each Cs^+^-sensitive mutant strain ranged from 1.2 × 10^−8^ to 5.4 × 10^−10^. In general, the reversion mutation rates are stable at 1.0 × 10^−8^ or less and are unstable at 1.0 × 10^−6^ or more, and all five mutant strains were phenotypically considered stable.

Compared with the wild type, strain Mut3 was observed to exhibit decreased growth, even in the absence of CsCl. In addition, strain Mut3 exhibited completely inhibited growth upon adding 100 mM CsCl to the medium. As with strain Mut3, strain Mut3R decreased growth, even in the absence of CsCl due to mutations in genes other than Cs^+^ resistance-related genes. The growth of Mut3R was inhibited by adding 800 mM Cs^+^ and Cs^+^ resistance of strain Mut3R was a partial recovery compared with the Cs^+^ resistance of the wild-type strain. Comparing and analyzing the mutation sites of strains Mut3 and Mut3R using the whole-genome sequence data, a mutation site difference of the MTS1_00475 gene region was observed. Therefore, MTS1_00475 was selected as a candidate for the Cs^+^ resistance-related genes.

There are two possibilities for the growth phenotypes of Mut3 and Mut3R. There are other Cs^+^ resistance-related genes where mutations may have occurred. For example, microorganisms are known to have large and diverse Cation/proton antiporters complementing adaptation to the external environment (Krulwich et al., 2009). Therefore, it is possible that strain TS-1 also has multiple Cs^+^-resistance mechanisms. Second, Cs^+^ resistance also decreased due to mutations in growth-related genes. From the Cs^+^ resistance growth test results, the growth of strain Mut3R reduced even without Cs^+^. Several studies have reported that mutations in the energy production system decrease growth and resistance to cations. A study by Hasim et al. (2018) reported that gene mutations in the energy-producing system that synthesize phosphatidylserine and phosphatidylethanolamine in *Candida albicans* reduced growth and resistance to farnesol. A study by Hamilton et al. (2002) also reported that the vacuolar H^+^-ATPase gene mutations of *Saccharomyces cerevisiae* showed poor growth and Na^+^ resistance. These suggest that strain Mut3R may also have gene mutations of the energy production system, resulting in decreased growth and Cs^+^ resistance. From the results of next-generation sequence analysis, 145 mutations were found in the strain Mut3. Therefore, revertant mutants with restored growth and Cs^+^ resistance will be obtained in future studies, and the reverted gene identified for further Cs^+^ resistance-related and gene candidates.

Mut4 grew at the same level as the wild strain in the absence of Cs^+^. However, growth was inhibited by the addition of 100 mM Cs^+^. Mut4R showed almost the same level of growth as the wild type, with or without Cs^+^. Therefore, this phenotype suggested that the revertant mutation site of Mut4R encodes a gene that is critical for Cs^+^ resistance. When the MTS1_00475 gene region of Mut4 and Mut4R strains were compared, it was confirmed that the mutation in MTS1_00475 was restored [W253*(stop codon) → *253R] by intragenic suppression. This result strongly suggests that MTS1_00475 is a Cs^+^ resistance-related gene. The mutation sites identified in Mut3 and Mut4 strains were 145 and 31, respectively. As strain Mut4 had fewer mutations than strain Mut3, this may have contributed to the recovery of the mutant Mut4R phenotype to a level that is almost the same as that of the wild type.

In this study, MTS1_00475 was identified as a Cs^+^ resistance-related protein by multiple Cs^+^-sensitive mutants and their revertant mutants. Strains Mut3 and Mut4 were first isolated, and the same gene mutations were identified in other Cs^+^-sensitive mutants (Mut6, Mut8, Mut9; Table 2).

When the MTS1_00475 gene region of strain Mut9R was compared, it was confirmed that the mutation of MTS1_00475 was restored (W177* → *177R) by intragenic suppression. This may be why Mut9R did not exhibit restored growth in response to high concentrations of CsCl, that is, to the same level as the wild type (Figure 4). Therefore, to discover novel genes related to Cs^+^ resistance, it is important to isolate a Cs^+^-sensitive mutant with a mutation other than the MTS1_00475 gene.

We will try to obtain MTS1_00475 by changing the screening medium in the future. However, since a mutation in MTS1_00475 isolates so many Cs^+^-sensitive strains, it is suggested that MTS1_00475 is a dominant Cs^+^ resistance mechanism.

### Functional Analysis of Cs^+^ Resistance-Related Gene Candidate MTS1_00475

Prediction of protein structure using TMHMM revealed that MTS1_00475 is a 14-transmembrane protein (Figure 6). The MTS1_00475 mutations in Mut3, Mut4, Mut8, and Mut9 were all mutated to the stop codon inserted in the first half of the MTS1_00475. As a result, MTS1_00475 did not function and showed Cs^+^ sensitivity. In addition, the strain Mut4R exhibited intragenic suppression of MTS1_00475-Trp253Arg. Nevertheless, Cs^+^ resistance was comparable to that of the wild type suggesting that this site did not significantly affect the function of MTS1_00475.

In the Cs^+^ resistance growth test, when the *MTS1_00475* gene was expressed in *E. coli*, no improvement in Cs^+^ resistance was observed. However, the Cs^+^/H^+^ antiport activity of MTS1_00475 was measured under high Cs^+^ conditions by producing everted membrane vesicles. In a previous report of K^+^/H^+^ antiporter activity using the right-side-out membrane of alkaliphilic *Bacillus* sp. No. 66, Rb^+^, and Cs^+^ were active as substrates (Kitada et al., 1997). However, no protein with Cs^+^/H^+^ antiporter activity has been reported. Therefore, MTS1_00475 was the first protein with Cs^+^/H^+^ antiporter (CshA) (MTS1_00475 is hereafter referred to as CshA).

As a future task, it is expected that the substrate affinity of CshA for Cs^+^ will be improved by introducing random mutations into the *cshA* gene using error-prone PCR.

Expression of a heterologous Na^+^/H^+^ antiporter gene in the *E. coli* strain KNabc lacking the three major Na^+^/H^+^ antiporter genes improve the cells Na^+^ resistance (Southworth et al., 2001; Liu et al., 2005; Morino et al., 2008). However, the expression of CshA did not improve Cs^+^ resistance in *E. coli*, probably due to the low affinity for Cs^+^ and sensitivity of *E. coli* to 100 mM CsCl. Subsequently, a host will confirm the improvement in Cs^+^ resistance by CshA with high Cs^+^ resistance. In addition, Cs^+^ resistance of the host will be improved by introducing a mutation into the *CshA* gene to produce a mutated CshA with a high affinity for Cs^+^.

It is inferred that the Cs^+^ resistance mechanism in strain TS-1 involves the consistent reduction of the intracellular Cs^+^ concentration by CshA in such a manner that the interior of the cell is not exposed to high concentrations of Cs^+^.

As a further functional analysis of CshA, the antiport activity of monovalent cations other than Cs^+^ was measured, and antiport activity against Na^+^, K^+^, and Rb^+^ other than Cs^+^ was observed (Supplementary Figure S2). As the apparent *K*_m_ value for these cations was approximately 1 mM, CshA was found to have a high affinity for Cs^+^. Strain TS-1 has seven genes annotated as Na^+^ (K^+^)/H^+^ antiporters, one belonged to the NhaA family (MST1_02247), one belonged to the NhaD-type Na^+^/H^+^ antiporter (MTS1_00585), two belonged to the NhaP-type Na^+^ (K^+^)/H^+^ antiporter (MTS1_01618, MTS1_03246), and three belonging to the CPA3 family (multi-subunit Na^+^/H^+^ antiporter) (MTS1_01874–MTS1_01879, MTS1_02182–MTS1_02187, MTS1_02374–MTS1_02381). These major Na^+^ (K^+^)/H^+^ antiporters are involved in Na^+^ and K^+^ homeostasis. Several microorganisms have multiple Na^+^ (K^+^)/H^+^ antiporters to adapt to different environments (Krulwich et al., 2009); however, there are no reports of antiporters belonging to these families with Cs^+^/H^+^ antiport activity. In addition, CshA was mainly identified in *Microbacterium* spp., isolated from various environments, such as dairy goods, oral cavities, and human clinical specimens (Tsuzukibashi et al., 2015). Phylogenetic tree analysis revealed that the source of bacteria harboring homologs having high homology with MTS1_00475 was the soil. As the strain TS-1 is a bacterium isolated from jumping spiders, it is suggested that strain TS-1 is closely related to the bacterium isolated from terrestrial soil. The physiological mechanisms by virtue of which *Microbacterium* sp. TS-1 has a high Cs^+^ resistance is still unknown.

Strain TS-1 is an alkaliphilic bacterium isolated from a ground jumping spider. Alkaliphilic bacteria were isolated from the posterior intestine of termites (Thongaram et al., 2005), beetle larvae intestines (Aizawa et al., 2010), and human feces (Vedder, 1934). The gut portion of termites that feed on soil generally contains large amounts of potassium ions, and their pH is highly alkaline (Bignell et al., 1983; Brune and Kuhl, 1996). Gut alkalinity promotes the solubilization and uptake of soil organic matter (Thongaram et al., 2003). In general, alkaliphilic bacteria isolated from soil require Na^+^ for growth. However, bacteria isolated from the above-described species require Na^+^ or K^+^ for growth (Horikoshi, 1991; Ito et al., 2011). If strain TS-1 acquired high-concentration Cs^+^ resistance in the process of growing in the special environment of the jumping spider, it would be interesting to elucidate the acquisition mechanism.

## Conclusion

We clarified the mechanism by which, in strain TS-1, intracellular Cs^+^ concentration is reduced by excreting Cs^+^ that has flowed into the cell at a high concentration to the outside of the cell by CshA. There have been no reports in the past that the homolog of MTS1_00475 has Cs^+^/H^+^ antiport activity. In addition, since this protein is widely conserved in bacteria belonging to the genus *Microbacterium*, it is a protein specific to this genus.

We obtained another Cs^+^-sensitive mutant from strain TS-1, and the existence of another Cs^+^ resistance mechanism was expected. There have been reports of isolation of high Cs^+^-resistant bacteria, but there are no reports that elucidate the mechanism of their Cs^+^ resistance. Therefore, this study is the first to elucidate the mechanisms of Cs^+^ resistance.

As a prospect, it is conceivable to express CshA in everted membrane vesicles and recover Cs^+^ in the membrane vesicles as a bioremediation tool, for example, for treating radioactive Cs^+^ contaminated water. CshA is a low-affinity Cs^+^/H^+^ antiporter. Therefore, by introducing mutations into CshA, it is necessary to produce a CshA mutant protein with a high affinity for Cs^+^. In the future, it will be necessary to establish a method for employing CshA in radioactive Cs^+^ recovery technology.

## Data Availability Statement

The datasets presented in this study can be found in online repositories. The names of the repository/repositories and accession number(s) can be found in the article/Supplementary Material.

## Author Contributions

MI: designed the research. TK, YI, YK, EI, MT, NM, KS, and MI: conducted the research. TK, YI, and MI: analyzed the data. MI: wrote the paper. All authors contributed to the article and approved the submitted version.

## Funding

This work was supported by a grant for the Toyo University Top Priority Research Promotion Program and the Toyo University intellectual property practical application promotion program.

## Conflict of Interest

The authors declare that the research was conducted in the absence of any commercial or financial relationships that could be construed as a potential conflict of interest.

## Publisher’s Note

All claims expressed in this article are solely those of the authors and do not necessarily represent those of their affiliated organizations, or those of the publisher, the editors and the reviewers. Any product that may be evaluated in this article, or claim that may be made by its manufacturer, is not guaranteed or endorsed by the publisher.

## Footnotes

^1^ https://www.geneius.de/GENEius/

^2^ https://blast.ncbi.nlm.nih.gov/Blast.cgi?PAGE=Proteins

^3^ https://mafft.cbrc.jp/alignment/server/

^4^ https://code.google.com/archive/p/treeviewx

^5^ https://services.healthtech.dtu.dk/service.php?TMHMM-2.0
