# Corrigendum: Novel Cesium Resistance Mechanism of Alkaliphilic Bacterium Isolated From Jumping Spider Ground Extract

**DOI:** 10.3389/fmicb.2022.902692

**Published:** 2022-05-04

**Authors:** Takahiro Koretsune, Yoshiki Ishida, Yuri Kaneda, Eri Ishiuchi, Miyu Teshima, Nanami Marubashi, Katsuya Satoh, Masahiro Ito

**Affiliations:** ^1^Graduate School of Life Sciences, Toyo University, Oura-gun, Japan; ^2^Faculty of Life Sciences, Toyo University, Oura-gun, Japan; ^3^Department of Radiation-Applied Biology Research, Takasaki Advanced Radiation Research Institute, Quantum Beam Science Research Directorate, National Institutes for Quantum Science and Technology, Takasaki, Japan; ^4^Bio-Nano Electronics Research Center, Toyo University, Kawagoe, Japan; ^5^Bio-Resilience Research Project (BRRP), Toyo University, Oura-gun, Japan

**Keywords:** cesium-resistant microorganisms, *Microbacterium*, alkaliphilic, mutant, whole-genome sequencing

In the original article, there was a mistake in Figure 7 as published. Figure 7 corresponds to Figure 8. The correct Figure 7 and Figure 8 and their legends have been posted, and the relevant part of the text has been revised. The corrected Figure 7 and Figure 8 and their legends appear below.

**Figure 7:**
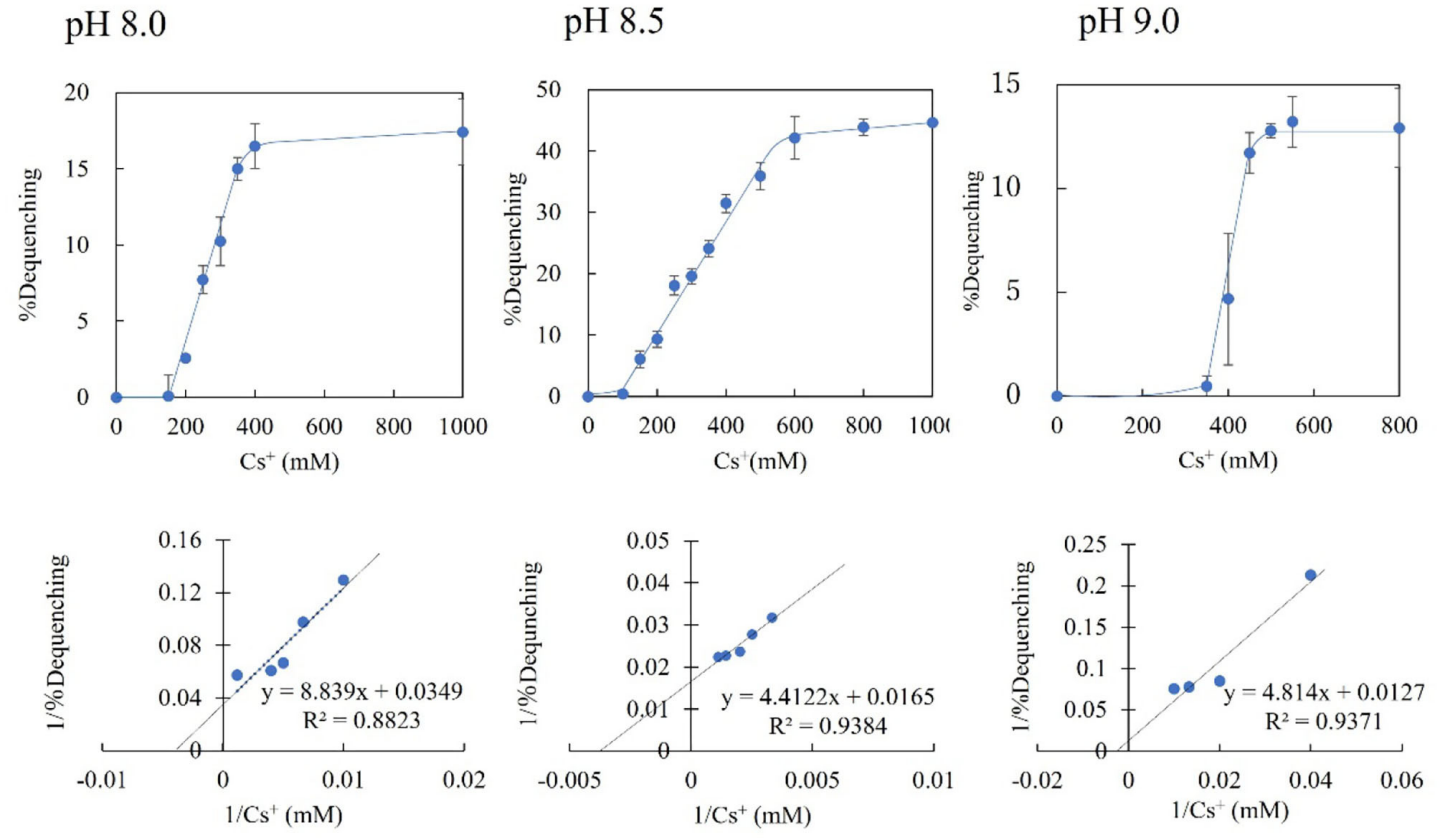
Cs^+^/H^+^ antiport activity of everted membrane vesicle from strain KNabc/pBAD-00475 at pH 8.0, 8.5, and 9.0. The Cs^+^/H^+^ antiport activity of everted membrane vesicles from strain KNabc/pBAD-00475 in each pH buffer was shown when various concentrations of Cs_2_SO_4_ were added. The details are described in the Materials and Methods section. The vertical axis shows antiport activity (%Dequenching), and the horizontal axis shows the Cs^+^ concentration. Error bars show the standard deviation of three independent experiments. In addition, each Lineweaver-Burk plot diagram is shown.

**Figure 8:**
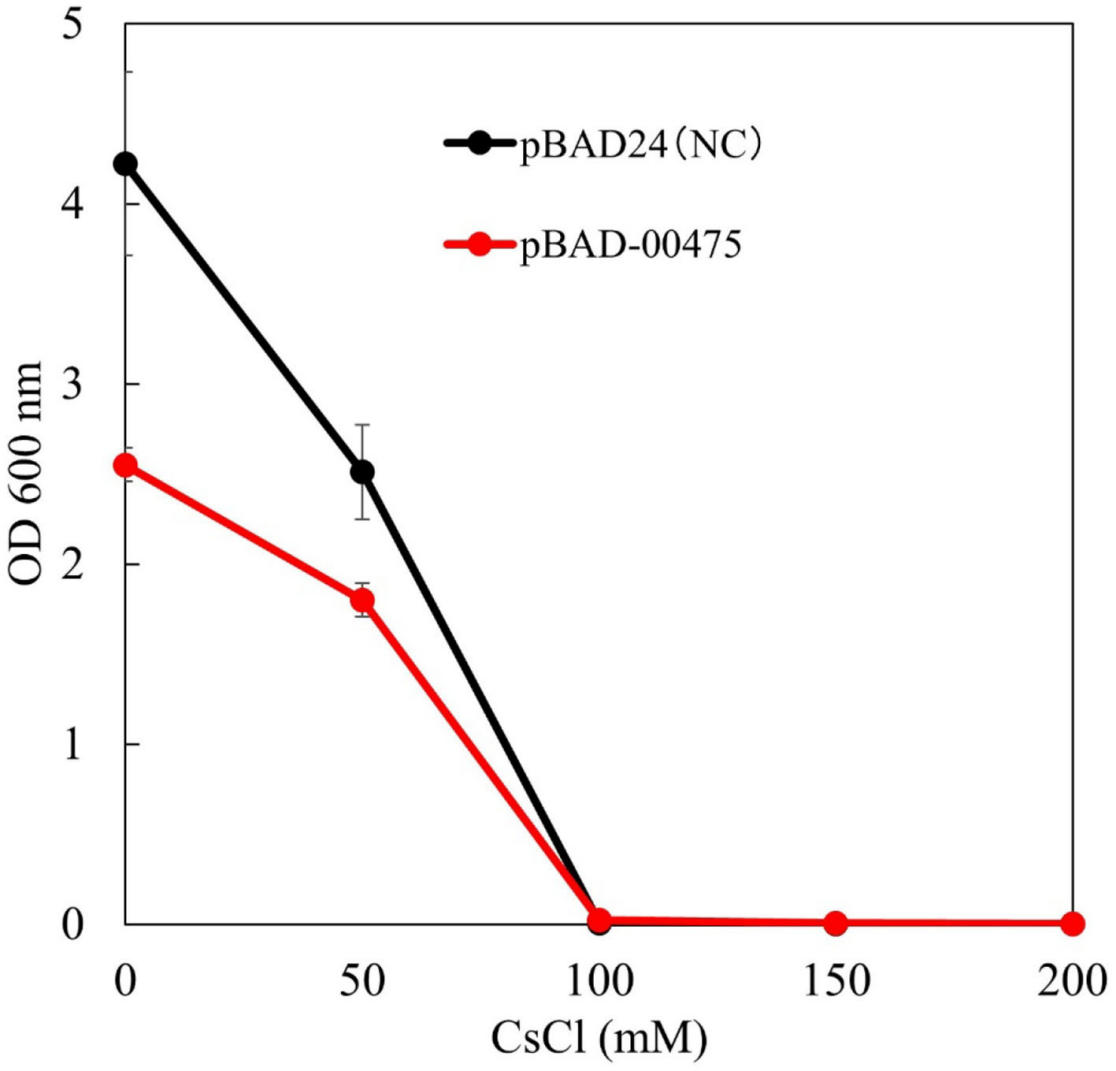
Test to evaluate the Cs^+^ resistance growth of strain KNabc/pBAD-00475. The turbidity of each concentration of CsCl when strain KNabc/pBAD-00475 in LBK medium was cultured for 16 h. Error bars show the standard deviation of three independent experiments. As the negative control, KNabc/pBAD24 was used.

In the original article, there was an error. A correction has therefore been made to the Results, sections **Cs**^**+**^ **resistance growth test of *E. coli* KNabc/pBAD-00475**. The corresponding figures are correctly associated in the text:

**Cs**^**+**^ **Resistance Growth Test of *E. coli* KNabc/pBAD-00475**

To confirm whether the Cs^+^ resistance of *E. coli* KNabc expressing MST1_00475 was improved, a Cs^+^ resistance growth test was conducted. Figure 8 shows the results of OD_600_ after independently culturing *E. coli* KNabc expressing MST1_00475 in a medium with different CsCl concentrations for 16 h. KNabc/pBAD-00475 and KNabc/pBAD24 (negative control) showed no growth in the presence of 100 mM CsCl. The expression of MTS1_00475 confirmed no improvement in Cs^+^ resistance. This result may be related to the low affinity of MTS1_00475 Cs^+^/H^+^ antiport activity.

The authors apologize for this error and state that this does not change the scientific conclusions of the article in any way. The original article has been updated.

## Publisher's Note

All claims expressed in this article are solely those of the authors and do not necessarily represent those of their affiliated organizations, or those of the publisher, the editors and the reviewers. Any product that may be evaluated in this article, or claim that may be made by its manufacturer, is not guaranteed or endorsed by the publisher.

