# Supplementary material for: Novel Cesium Resistance Mechanism of Alkaliphilic Bacterium Isolated From Jumping Spider Ground Extract
Source: Front Microbiol. 2022 Mar 8;13:841821. doi: 10.3389/fmicb.2022.841821 (PMC8984678; doi:10.3389/fmicb.2022.841821)
Supplement: Supplementary file 1 [file Table_1.DOCX]

Additional file 1

Novel cesium resistance mechanism of alkaliphilic bacteria isolated from jumping spider ground extract

**Takahiro Koretsune^1^, Yoshiki Ishida^1^, Yuri Kaneda^2^, Eri Ishiuchi^2^, Miyu Teshima^2^, Nanami Marubashi^2^, Katsuya Sato^3^ and Masahiro Ito^1,2,4,5*^**

^1^Graduate School of Life Sciences, Toyo University, Oura-gun, Gunma 374-0193 Japan

^2^Faculty of Life Sciences, Toyo University, Oura-gun, Gunma 374-0193 Japan

^3^Department of Radiation-Applied Biology Research, Takasaki Advanced Radiation Research Institute, Quantum Beam Science Research Directorate, National Institutes for Quantum and Radiological Science and Technology, Takasaki, Gunma, Japan

^4^Bio-Resilience Research Center, Toyo University, Oura-gun, Gunma 374-0193 Japan

^5^Bio-resilience research project (BRRP), Toyo University, Oura-gun, Gunma 374-0193 Japan


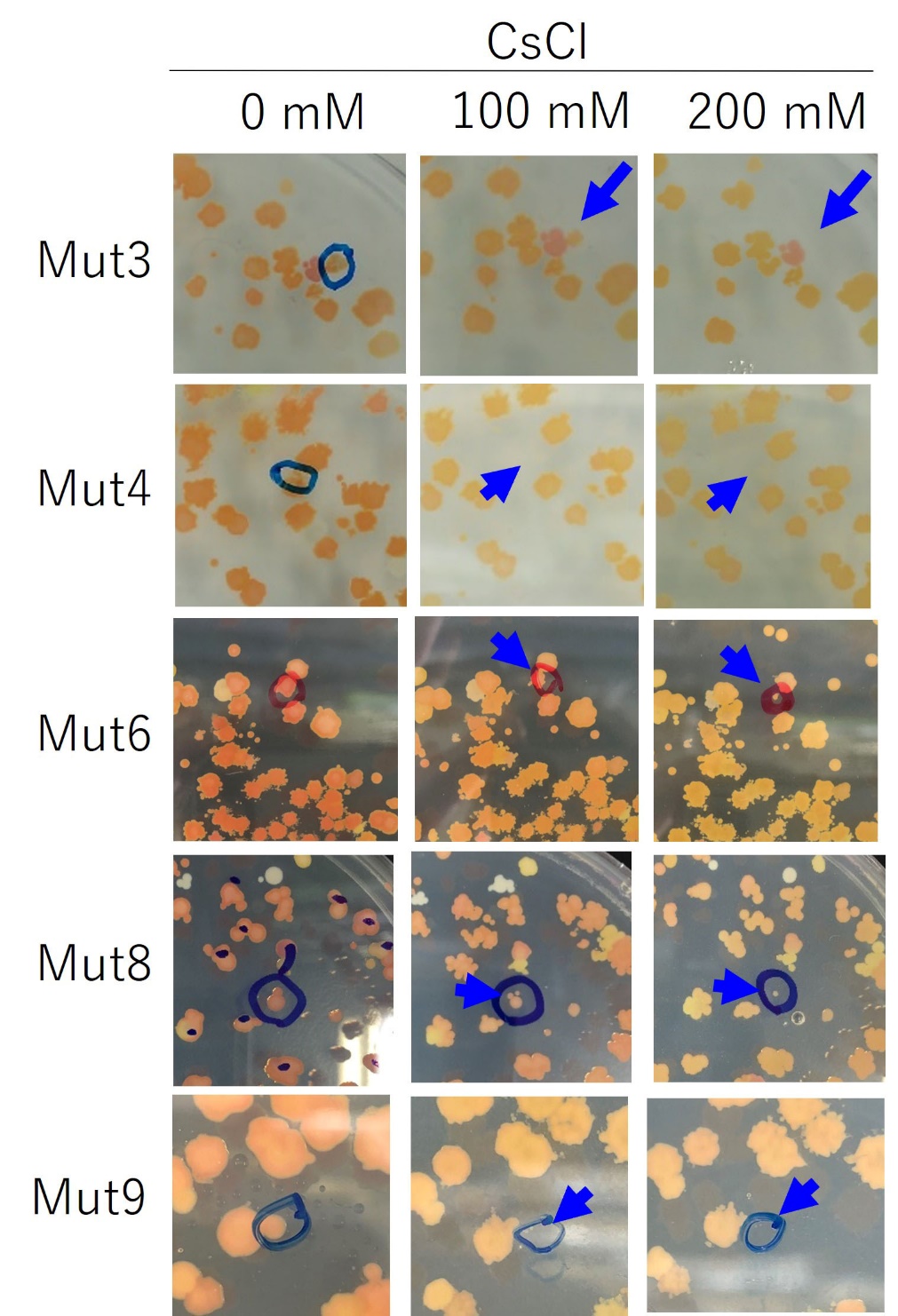


**Supplementary Figure S1. Isolation of Cesium ion-sensitive mutants by replica plating method**

The colonies of cesium ion-sensitive mutants obtained by chemical mutation treatment by the replica plating method are shown. From the left column, CsCl-free NC agar medium, 100 mM CsCl-containing NC agar medium, and 200 mM CsCl-containing NC agar medium, the pH of each medium is 8.0. Blue arrows indicate colonies that were sensitive to CsCl.


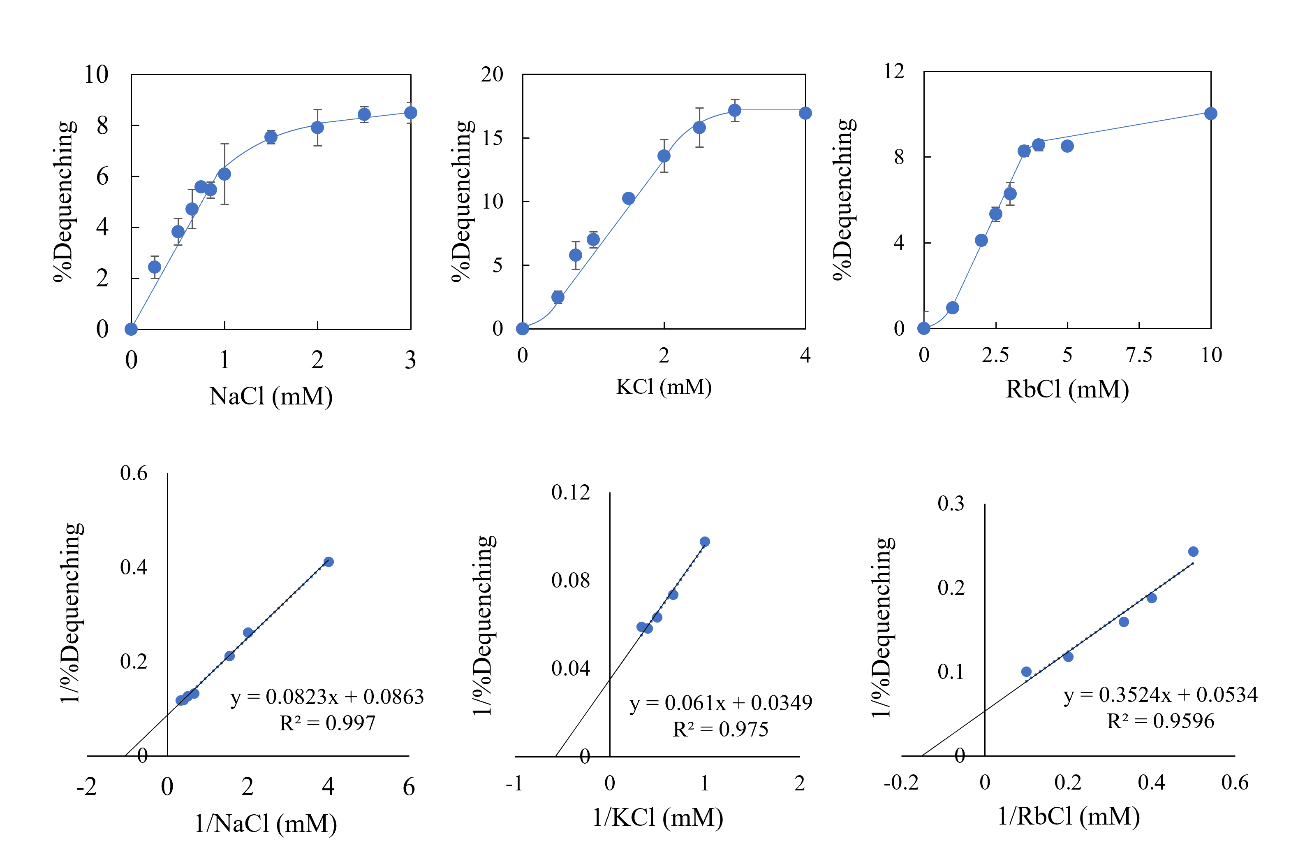
**Supplementary Figure S2. Cation/H^+^ antiport activity of everted membrane vesicle from strain *E. coli* KNabc/pBAD-00475 at pH 8.0**

The cation/H^+^ antiport activity of everted membrane vesicle from strain *E. coli* KNabc/pBAD-00475 at pH 8.0 was shown when various concentrations of each cation was added. The detail was described in Materials and methos section. The vertical axis shows antiport activity (%Dequenching) and the horizontal axis shows each cation concentration. The error bars show the standard deviation for three independent experiments. In addition, each Lineweaver-Burk plot diagram is shown.
